# Changes in Hepatic Hemodynamics due to Primary Liver Tumours

**DOI:** 10.1155/1996/62057

**Published:** 1996

**Authors:** F. Jakab, Z. Ráth, F. Schmal, P. Nagy, J. Faller

**Affiliations:** Department of Surgery Semmelweis University of Medicine & St. John Hospital Budapest Hungary Supported by a grant from Ministry of Welfare M-005/1990 Hungary

## Abstract

Data regarding the afferent circulation of the liver in patients with primary hepatocellular carcinoma are controversial, we have carried out measurement of hepatic arterial and portal venous flow intraoperatively by transit time ultrasonic volume flowmetry. In patients with primary hepatocellular carcinoma the hepatic artery flow increased to 0.55±0.211 compared with the control value of 0.37±0.102 1/min. (p<0.01). The portal venous flow decreased from 0.61±0.212 l/min, to 0.47±l/min. p<0.01). Due to the opposite changes in the afferent circulation the total hepatic blood flow did not change significantly, compared with controls.

The ratio of hepatic arterial flow to portal vein flow increased to 1.239±0.246 in patients with hepatocellular carcinoma, which is double of the control value (0.66±0.259 l/min). After resection this ratio did not change.

The resection did not alter hepatic artery or portal venous flow significantly, although the total hepatic blood flow decreased significantly (p<0.01).

On the basis of our early results it is possible that the ratio of the two circulations may be to deel measured with doppler ultrasound and provide diagnostic information.


*HPB Surgery*, 1996, Vol.9, pp.245–248
Reprints available directly from the publisher
Photocopying permitted by license only

© 1996 OPA (Overseas Publishers Association)
Amsterdam B.V. Published in The Netherlands
by Harwood Academic Publishers GmbH
Printed in Malaysia

### Changes in Hepatic Hemodynamics due to Primary Liver Tumours

F. JAKAB, Z. RÁTH, F. SCHMAL, P. NAGY and J. FALLER

Department of Surgery, Semmelweis University of Medicine & St. John Hospital Budapest, Hungary
Supported by a grant from Ministry of Welfare, HUNGARY M-005/1990

*(Received 28 December 1993)*

Data regarding the afferent circulation of the liver in patients with primary hepatocellular carcinoma are controversial, we have carried out measurement of hepatic arterial and portal venous flow intraoperatively by transit time ultrasonic volume flowmetry. In patients with primary hepatocellular carcinoma the hepatic artery flow increased to 0.55±0.211 compared with the control value of 0.37±0.102 l/min. ($p<0.01$). The portal venous flow decreased from 0.61±0.212 l/min. to 0.47±l/min. $p<0.01$). Due to the opposite changes in the afferent circulation the total hepatic blood flow did not change significantly, compared with controls.

The ratio of hepatic arterial flow to portal vein flow increased to 1.239±0.246 in patients with hepatocellular carcinoma, which is double of the control value (0.66±0.259 l/min). After resection this ratio did not change.

The resection did not alter hepatic artery or portal venous flow significantly, although the total hepatic blood flow decreased significantly ($p<0.01$).

On the basis of our early results it is possible that the ratio of the two circulations may be to deel measured with doppler ultrasound and provide diagnostic information.

KEY WORDS: Primary hepatocellular carcinoma afferent circulation of the liver portal venous flow hepatic arterial flow

### INTRODUCTION

Many detials of the vascular patterns in liver tumors have been clarified, however the effect of liver tumors on afferent hepatic circulation is a porrly investigated field. In this study the afferent liver circulation was measured in patients with hepatocellular carcinoma. The hepatic arterial flow and portal venous flow were measured before, and after resection, although it is said that partial liver resection produces little change in total liver blood flow[15,16,17]. This occurs because the major contributor to total flow, the portal vein is affected less by events taking place within the liver than by control mechanisms in the arterial resistance vessels of the prehepatic splanchnic bed. As the total blood flow is therefore redistributed to a smaller mass of liver tissue on increase in ml/min/unit tissue weight could be expected in the non-resected part of the liver.

In this study the effect of primary liver tumors and of resection were investigated on afferent hepatic circulation using ultrasonic transit time volume flowmeter. (Transonic System Inc.)

### MATERIAL AND METHODS

Fifteen patients with a mean age of 46.5 years (range 28 to 69) underwent abdominal surgical exploration for hepatocellular carcinoma.

Resection of the tumour was carried out in every case. The informed consent for the circulatory studies was obtained from all patients. All interventions were carried out under general anesthesia with endotracheal intubation.

The blood pressure, blood pH, blood gases, $O_2$ saturation were monitored carefully and urine output. Patients were only included in if study if the changes of blood pressure did not exceed 10% of the basic values, urine output was steady, and there was no significant blood loss. After having explored

*Correspondence to*: Ferenc Jakab M.D.Ph.D. Professor of Surgery Budapest, Uzsoki street 2p. HUNGARY, H-1145 Fax/Phone: 36-1 156-3049

the abdominal cavity and decided resection was possible the HA and PV were isolated for security reasons.

The flow probes were placed on the HA and the PV after determination of electronic zero. The diameter of flow probes corresponded to the diameter of the vessels. The simultaneous measurements of hepatic artery flow (HAF) and portal venous flow (PVF) were obtained by means of a transit time ultrasonic volume flowmeter (Model HT 207, Transonic System inc., Ithaca, N.Y., U.S.A.)

After registration of basal values of HAF and PVF the necessary resection was carried out using CUSA (Valleylab). Having finished the resection the measurement of HA and PV was repeated, and recorded.

The mean ± standard error were calculated. Statistical significance of the changes was assessed with Student'test applied to the differences between the control and observed values. (Mattheas Program)

### RESULTS

In fifteen patients with hepatocellular carcinoma the average value for hepatic artery flow (HAF) was 0.55±0.211 l/min. compared with the control value of 0.377±0.11 l/min. ($p < 0.01$). These latter values were from patients without liver cancer.[11] In the same (patients) the portal venous flow (PVF) was 0.61±0.212 l/min and 0.47±0.203 l/min respectively ($p < 0.01$) Figure 1 and Table 1. Due to these opposite changes, total hepatic inflow (THBF) was similar.

Arterial blood pressure was 143±5 mmHg and it was kept constant through out the procedure. In the afferent circulation in control patients, the HA contributed 37% and the PV 63% to the THBF. These results were in agreement with the values published in the literature.

The ratio of hepatic arterial flow (HAF) to portal venous flow (PVF) was 1.24±0.246 in patients with hepatocellular carcinoma which is double the control value (0.66±0.259; $p < 0.01$). After resection this ratio did not change ($p > 0.2$) Figure 2.

Resection of the liver did not alter the HAF or PVF, but the THBF decreased significantly ($p < 0.01$) Figure 3.

---

*Abbreviations:* HAF:hepatic arterial flow PVF:portal venous flow THBF:total hepatic blood flow HA: hepatic artery PV:portal vein

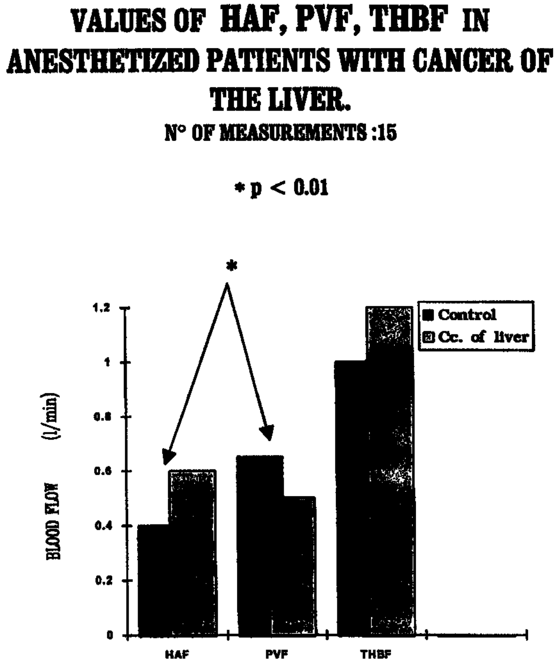

**Figure 1** Values of HAF, PVF, THBF in anasthetized patients with cancer of the liver

### DISCUSSION

The hepatic circulation is both large and complex. The blood flow in liver tumors and the circulatory changes caused by liver malignancy is of much interest in surgical practice. Both primary and secondary liver tumors are supplied chiefly by blood from the hepatic artery shown by Segall[20]. Ackerman[1] has measured the relative hepatic arterial and portal venous blood flow with microsphere techniques in liver tumors of different sizes. Small liver tumors receive their blood flow from both the hepatic artery and p ortal vein, but with most from the hepatic artery Acher[2]. It has been demonstrated by arteriography that well differentiated hepatocellular carcinomas are more vascular than poorly differentiated tumours. In the poorly

**Table 1** Values of the afferent hepatic circulation of patients with hepatocellular carcinoma

| | *HAF* | *PVF (l/min)* | *THBF* |
|---|---|---|---|
| CONTROL | 0.378* | 0.614 | 0.993 |
| N=14 | ±0.102 | ±0.212 | ±0.276 |
| Pts with | 0.549 | 0.467 | 1.016 |
| HCC N=15 | ±0.211 | ±0.203 | ±0.410 |
| AFTER | 0.451 | 0.349 | 0.800 |
| RESECTION N=15 | ±0.216 | ±0.154 | ±0.359 |

*Mean ± SD

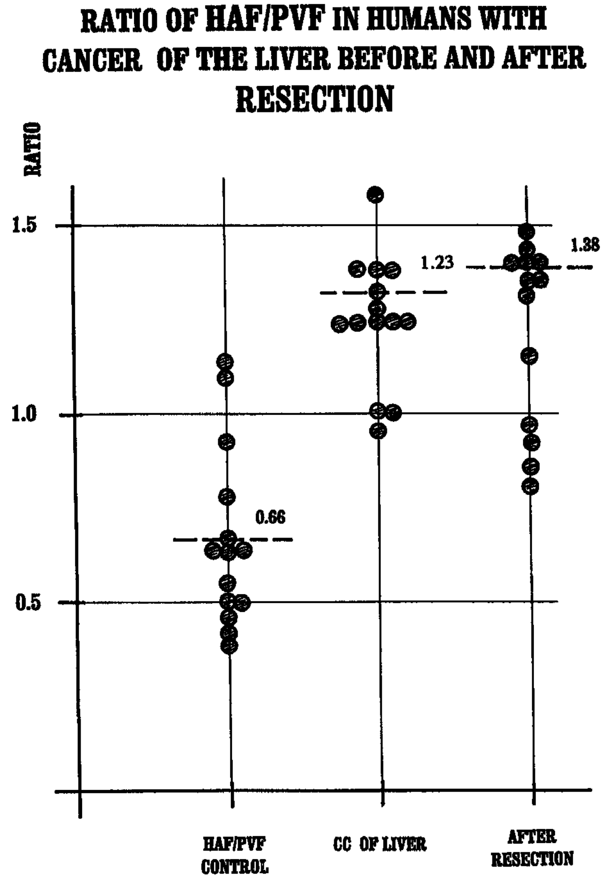

Figure 2 Ratio of HAF/PVF in humans with cancer of the liver before and after resection differentiated tumors the portal vessels were chiefly localized in the peripheral part. The relationship between tumor size and vascular pattern has been investigated by Carlsson[5] with repeated hepatic arteriography and portography, he reported that large tumors had a vascular pattern predominantly arterial or predominantly portal or a combination of the two.

Most likely haemodynamic abnormalities associated with hepato-cellular carcinoma can be explained by the alterations of portal venous flow caused by compression, infiltration, tumor invasion or associated thrombosis of the branches of theportal vein.

In our study the most striking circulatory alterations were as follows: the significant increase of HAF, and the significant decrease of PVF. The decrease of the venous flow can be attributed to the HCC.

Many consider the decrease of PVF as the primary circulatory change, which consequently causes increase in HAF[6,9,–14,21,22]. The explanation for the interaction of two afferent circulatory systems, and has been clarified by the extensive studies of Lautt and his coworkers[6,12–14].

The ratio of HAF/PVF is doubled in patients with HCC, and it seems likely, that the altered ratio has not only pathophysiological interest but it can also be used as a diagnostic tool. Having reviewed the literature similar observation have not been published.

This phenomenon might be useful for Doppler US diagnosis, e.g. in case of hypoechoic or hyperechoic mass in the liver and of decreased PVF, and of increased HAF a malignant tumor may be suspected.

Resection did not have a marked effect on the ratio of HAF to PVF or THBF and these data are consistent with the literature[8,15]. The major contributor to THBF, the PV is affected less by resection taking place within the liver than by control mechanisms in the splunchnir arterial resistance vessels. Because essentially the same total blood flow is redistributed to a smaller mass of liver tissue perfusion (ml/min/unit tissue weight) would be anticipated in the non-resected remnant.

In tissue perfusion studies it has been shown that patients with an obstructed portal vein exhibited little change in hepatic tissue perfusion after resection indicating the presence of maximal perfusion of liver in case of PV obstruction.[15].

The hepatic regeneration of the normal liver remnant proceeds rapidly following partial hepatic resection, major circulatory change can not be expected later on[8,15].

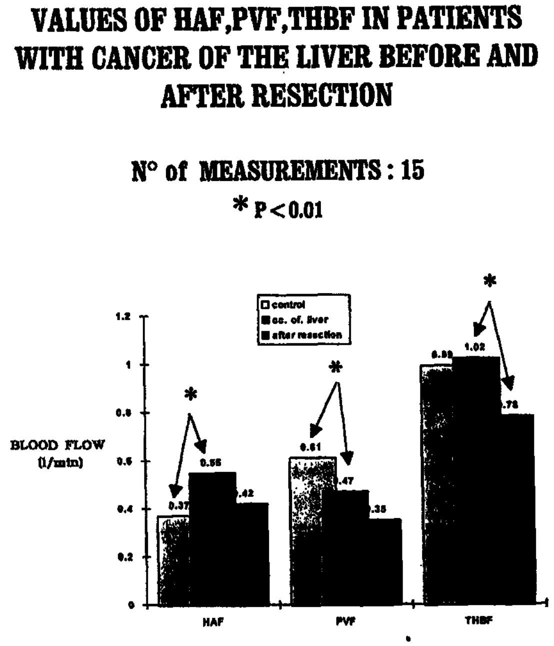

Figure 3 Values of HAF, PVF, THBF in patients with cancer of the liver before and after resection.

Finally one common event should be discussed on the basis of the circulatory data gained in patients with HCC. The primary circulatory change-is a decrease in PVF. The decrease of PVF does not seem to be uniquely characterising the HCC, or any other space occupying lesion. By the occlusion of the common bile duct others and ourselves found a significant primary decrease in PVF and subsequent increase in HAF [16.17,21,22]. In the patients with Klatskin tumors the decrease of PVF and increase of HAF have been observed (10) and finally there are numerous circulatory studies on cirrhotics showing a decrease of PVF, and secondary increase of HAF[3,4].

Based on our observations it seems reasonable and explicable, that all kinds of pathology (diffuse and nodulor space occupying) lead to a primary decrease in PVF, and subsequently to an increase in HAF. The theoretical explanation of this observation is that all circulatory change can be explained by the adenosin washout theory Lault[6,12,13,14].

### REFERENCES

1. Ackerman N.B.: (1986) Experimental Studies on the Role of the Portal Circulation in Hepatic Tumor Vascularity. *Cancer* **58**: 1653–1657.
2. Archer S.G., B.N. Gray: (1989) Vascularisation of Small Liver Metastasis. *Br. J. Surg.* **76**: 545–548.
3. Benoit J. N., Granger D.N.: (1993) Splanchnic Hemodynamics in Chronic Portal Hypertension Semin. *Liver Dis.* **6**: 287. 1986.
4. Blendis L. M.: Circulation in Liver Disease. *Transplant Proceedings* **25**: 174 1–43.
5. Carlsson G., Ekelund L., Hafström L. *et al.*: (1981) Effects of HA Ligation and Intraarterial Embolisation on Liver Tumor Growth. *Journal of Surgical Oncology*, **17**: 249–261.
6. Ezzat R.W., Lautt W.W.: (1987) Hepatic Arterial Pressure Flow Autoregulation is Adenosine Mediated. *Am. J. Physiol.* **252**: H. 836–845.
7. Greenway C.V., Stark R.D.: (1971) Hepatic Vascular Bed. *Physiol Rev* **51**: 23–65.
8. Hanna S.S.: (1988) Liver Blood Flow after Major Hepatic Resection. *Can. J. Surg.* **31**: 363–367.
9. Hendersson J.M., Gilmore G. Th., mackay Gr. J. *et al.*: (1992) Hemodinamics During Liver Transplantation: The Interaction between Cardiac Output and Portal Venous and Hepatic Arterial Flows. *Hepatology*. **16**: 715–718.
10. F. Jakab, T. Hernàdi: (1990)Changes in Hepatic Blood Flow in Jandice Due to Hilar carcinomas, the So-called Klastskin Tumours. *Acta Chirurgica Hungarica* **31**:247–253.
11. F. Jakab, Z, Ràth, F. Schmal, P. Nagy, J. Faller: (1994) The Interaciton Between Hepatic Arterial and Portal Venous Blood Flows: Simultaneous measurement by Transit Time Ultrasonic Volume Flowmetry. *J. Hepato-Gastroenterology*. Submitted for Publication .
12. Lautt W.W.: (1985) Mechanism and Role of Intrinsic Regulation of Hepatic Arterial Blood Flow: Hepatic Arterial Buffer Response. *Am.J. Physiol* **249**: G 549–556.
13. Lautt W.W.: (1985)Adenosine as Putative Regulator of Hepatic Arterial Flow. *Am. J. Physiol.* **248**: H. 331–338.
14. Lautt W.W., Legare D.J., Ezzat W.R.: (1990) Quantitation of the Hepatic Arterial Buffer Response to Graded Changes in Portal Blood Flow. *Gastroenterology* **98**: 1024–1028.
15. Mathie R.T., Blumgart L.H.: (1982) Tissue perfusion Studies during Partial Hepatectomy in Man Surgical. *Gastroenterology* **1**: 297–302.
16. Mathie R.T., Nagorney D.M., Lewis M.H., Blumgart L.H.: (1988) Hepatic Hemodynamics after Chronic Obstruction of the Biliary Tract in the Dog. *Surg. Gyn. Obstet.* **166**:125–130.
17. Nagorney D.M., Mathie R.T., Lygidakis N.J., Blumgart L.H.: (1982) Bile Duct Pressure as a Modulator of Liver Blood Flow after Common Bile Duct Obstruction. *Surg. Form.* **33**: 206–208.
18. Nilsson L.A.V., Zettergreen L.: (1967) Blood Supply and Vascular Pattern of Induced Primary Hepatic Carcinoma in Rats. *Acta pathologica Microbiologica Scand* **71**: 179–186.
19. Rice G.C., Leiberman D.P., Mathie R.T. *et al.*: (1977) Liver Tissue Blood Flow Measured by 85 Kr. Clearence in the Anesthetized Rat before and after partial Hepatectomy. *British Journal of Experimental Pathology* **58**: 243–250.
20. Segall H.N.: (1923) An Experimental Anatomical Investigation of the Blood and Bile Channels of the Liver. *Surgery, Gynecology and Obstetrics* **37**: 152-178.
21. G. Szabó F. Jakab, Z. Magyar: (1974) Effect of Acute Cholestasis on Hepatic Circulation. *Acta Med. Acad. Sci. Hung.* **31**: 229–239.
22. G. Szabó, F. Jakab, Z. Magyar: (1974) The Mechanisms of the Effect of Increased Biliary Pressure on Hepatic Circulation. *Acta Med. Acad. Sci. Hung.* **31**: 241–250.

